# Supplementary material for: Potential short-term negative versus positive effects of olive mill-derived biochar on nutrient availability in a calcareous loamy sand soil
Source: PLoS One. 2020 Jul 2;15(7):e0232811. doi: 10.1371/journal.pone.0232811 (PMC7332016; doi:10.1371/journal.pone.0232811)
Supplement: S1 Fig — (DOCX) [file pone.0232811.s001.docx]

Fig. S1. FTIR spectra of the produced olive mill solid waste-derived biochar (OMSW-BCs) (BC300: biochar produced at 300 °C; BC400: biochar produced at 400 °C; BC500: biochar produced at 500 °C; BC600: biochar produced at 600 °C; BC700: biochar produced at 700 °C).
