# Supplementary material for: Potential short-term negative versus positive effects of olive mill-derived biochar on nutrient availability in a calcareous loamy sand soil
Source: PLoS One. 2020 Jul 2;15(7):e0232811. doi: 10.1371/journal.pone.0232811 (PMC7332016; doi:10.1371/journal.pone.0232811)
Supplement: S3 Fig — (DOCX) [file pone.0232811.s003.docx]

Fig. S3. Scanning electron microscope (SEM) analyses of feedstock (FS) and olive mill solid waste-derived biochars (OMSW-BCs) pyrolyzed at different temperatures (a: FS: feedstock; b: BC300: biochar produced at 300 ^o^C; c: BC400: biochar produced at 400 ^o^C; d: BC500: biochar produced at 500 ^o^C; e: BC600: biochar produced at 600 ^o^C; f: BC700: biochar produced at 700 ^o^C).
